# The importance of visual control and biomechanics in the regulation of gesture-speech synchrony for an individual deprived of proprioceptive feedback of body position.

**DOI:** 10.1038/s41598-022-18300-x

**Published:** 2022-08-30

**Authors:** Wim Pouw, Steven J. Harrison, James A. Dixon

**Affiliations:** 1 Center for the Ecological Study of Perception and Action, University of Connecticut, Storrs, USA; 2 Donders Institute for Brain, Cognition and Behaviour, Radboud University Nijmegen, Nijmegen, The Netherlands; 3 Max Planck Institute for Psycholinguistics, Nijmegen, The Netherlands; 4 Department of Kinesiology, University of Connecticut, Storrs, USA; 5 Department of Psychological Sciences, University of Connecticut, Storrs, USA

**Keywords:** Neuroscience, Psychology

## Abstract

Do communicative actions such as gestures fundamentally differ in their control mechanisms from other actions? Evidence for such fundamental differences comes from a classic gesture-speech coordination experiment performed with a person (IW) with deafferentation (McNeill, 2005). Although IW has lost both his primary source of information about body position (i.e., proprioception) and discriminative touch from the neck down, his gesture-speech coordination has been reported to be largely unaffected, even if his vision is blocked. This is surprising because, without vision, his object-directed actions almost completely break down. We examine the hypothesis that IW’s gesture-speech coordination is supported by the biomechanical effects of gesturing on head posture and speech. We find that when vision is blocked, there are micro-scale increases in gesture-speech timing variability, consistent with IW’s reported experience that gesturing is difficult without vision. Supporting the hypothesis that IW exploits biomechanical consequences of the act of gesturing, we find that: (1) gestures with larger physical impulses co-occur with greater head movement, (2) gesture-speech synchrony relates to larger gesture-concurrent head movements (i.e. for bimanual gestures), (3) when vision is blocked, gestures generate more physical impulse, and (4) moments of acoustic prominence couple more with peaks of physical impulse when vision is blocked. It can be concluded that IW’s gesturing ability is not based on a specialized language-based feedforward control as originally concluded from previous research, but is still dependent on a varied means of recurrent feedback from the body.

## Introduction

We can intuitively distinguish different kinds of actions in which humans engage. One broad distinction identifies actions that are directed towards objects, such as evading a candle to grasp a cup, or catching a moving ball. Such *object-directed actions* seem to differ from actions that are performed to communicate to other persons. Such *communicative actions* include speaking, manual signing, and manual gesturing during speaking. The distinction between object-directed and communicative actions has obvious face validity at the behavioral level, based on the presence or absence of a communicative goal. But what basis is there to distinguish these types of action in terms of their underlying motor control? For example, humans move their hands (a) to act on objects and (b) to communicate when gesturing while speaking. Are these two types of hand movements controlled by different systems? This is an important question because it has implications for whether human communication is based on uniquely specialized mechanisms only operable in communication or whether it is grounded in more domain-general mechanisms that are re-assembled in novel task-specific ways^1,2^.

Research on communicative manual actions during speaking (henceforth *gestures*) shows that they tend to be coordinated with speaking^3^. For example, gestures that depict objects are timed to synchronize with the portion of the speech that refers to what is depicted. Conversely, object-directed actions do not synchronize with the parts of the speech that refer to those actions^4^. Thus the performance variables in communicative actions versus object-directed actions are different in terms of the degree of coordination with speech. Even the means of acquisition may be different for communicative actions. For example, congenitally blind adults and children putatively gesture similarly as sighted persons^5,6^, which suggests that gestures are not learned through imitation, in contrast to many object-directed actions that are learned in this way (e.g., tying one's shoe laces). But some researchers have gone even further, suggesting to have found evidence for “the existence of a specific thought-language-hand link in the brain, distinct at some point from the pathways for controlling actions”^7^ (p. 236). This conclusion was drawn from an important case study of a person known as IW who lost the sense of his body movements and position (proprioception) from the neck down, but still seemed to synchronize his gestures normally with speech without any visual guidance^7–10^. Since IW’s action routines break down without visual guidance^11^, but gesture and speech remain coordinated^7^, communicative gesture-speech coordination may be entirely differently controlled than instrumental action. In this paper, we revisit this foundational experiment and reanalyze the original data with current-day methods. We hope to increase our understanding of how gesture is performed when feedback from the body is limited by addressing the following questions: Do gestures require proprioception to synchronize with speech? Is visual control important for gesturing when proprioception is absent? Are there other control loops that allow gesture and speech to synchronize, such as a biomechanical link between gesture and speech^12^? Answering these specific questions will have implications for the general theoretical question of whether manual communicative actions are grounded in domain-general motor control mechanisms or whether communicative actions are indeed “distinct at some point from the pathways for controlling actions” (McNeill^7^, p. 236).

### The loss of proprioception

Arguably no single case study has had such a lasting imprint on students of human movement, physiology, and multimodal language as that of IW^8–11^. IW’s perception of his body’s self-relative position in space (proprioception) and touch is compromised below the neck by a nerve-debilitating disease that struck during early adulthood^13,14^. Specifically, due to a virus, specific nerve fibers important for muscular feedback of actions degenerated. Consequently, in the absence of sight of his body, IW cannot directly perceive how his trunk and limbs are positioned and does not have discriminative touch. As far as is currently known, IW’s sense of temperature and muscle fatigue remain intact, and such pathways provide some information about body position, albeit minimally (Cole, 2016). Pain perception and deep touch has recently been found to be impaired in IW however^15,16^, which suggests that thickly myelinated A β nerves that are degenerated in IW may be important for pain and pressure perception. The loss of proprioception is a rarely documented condition, known to occur in only a few individuals worldwide and has therefore garnered attention from a diverse collection of researchers interested in the role of this body sense in neuropsychological functioning^9,17,18^.

IW’s case has served as an impressive demonstration for students of human movement^19^ in showing that successful performance of actions such as postural control, locomotion, and manual interactions with objects, requires the continuous perception of the evolving consequences of those actions^20^. Because IW does not have access to proprioceptive information about his body position, he must use the visual, auditory, and vestibular consequences of his actions. Early in IW’s disease such secondary feedback was not sufficient to help him control his movements. Any movement IW made created a risk of falling, due to the lack of anticipatory and reactive postural control. However, IW has served as an equally impressive demonstration of the human spirit^11^; He has relearned essential instrumental routines (e.g., walking, grasping) through visually-guided and cognitively intensive control of his actions. Thus, IW has been able to reorganize the action-perception loop by compensating for the lack of proprioception with the continuous visual monitoring of his actions. Without vision, object-directed actions create a risk of falling, unless he has countermeasures in place, such as stiffening the body or manually holding onto nearby support^11^.

**Figure 1:**
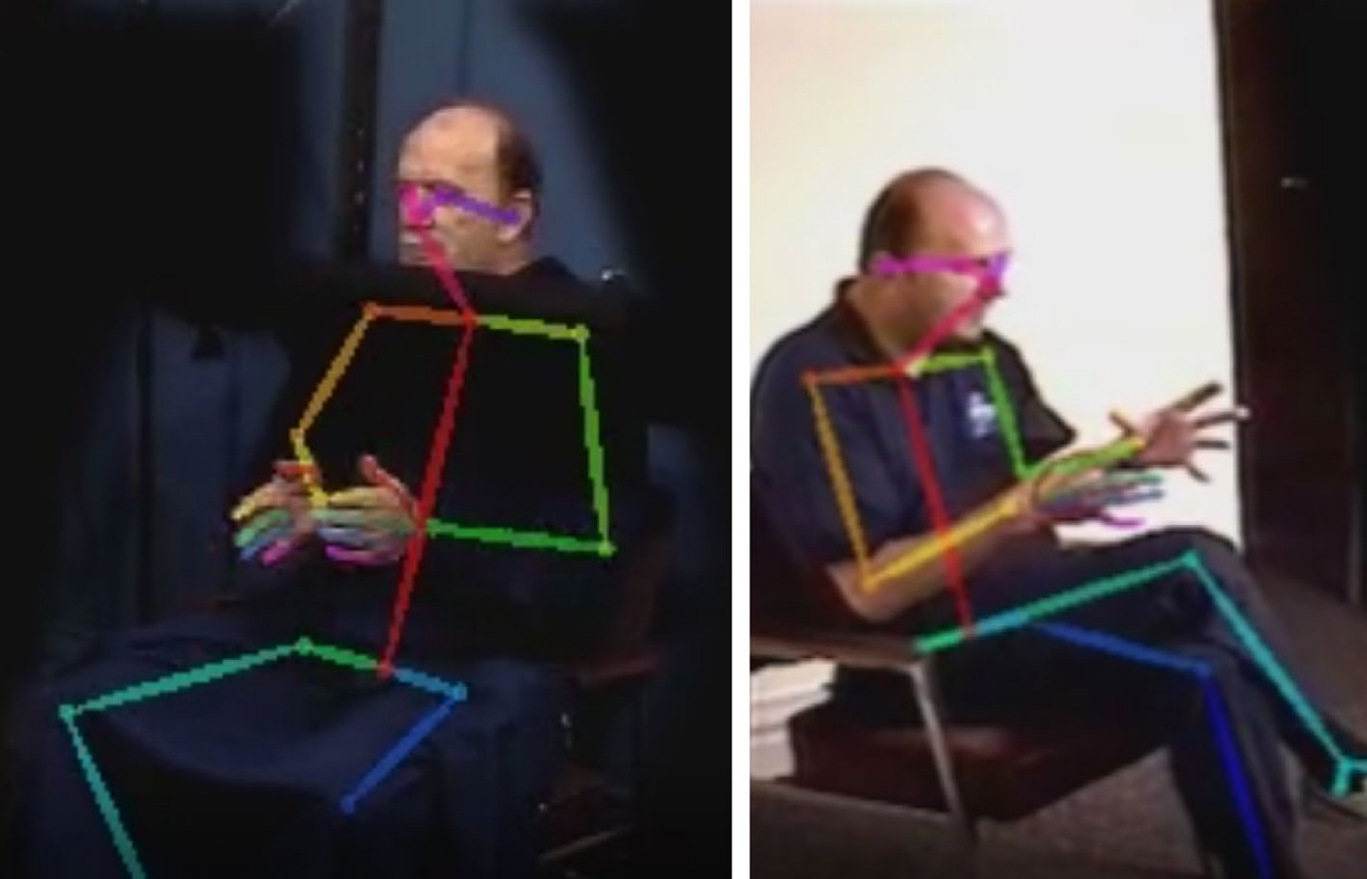
Example pose estimation from video-recordings. *Note*. Example of the 2002 (left panel; blind condition) and 1998 (right panel; no blind condition) experiment wherein we superimposed pose tracking skeleton extracted with OpenPose.

Remarkably, however, IW can produce typical communicative gestures during speech without visual feedback in ways that are nearly indistinguishable from the gestures IW makes *with* vision. This was reported in two experiments performed in 1998 and 2002 by gesture researcher David McNeill in collaboration with philosopher Shaun Gallagher and the physician-researcher Jonathan Cole^7–10,21^. In these experiments, IW was asked to retell a Tweety and Sylvester cartoon to evoke gesture and speech. In some retellings, IW’s vision of his hands was blocked by the presence of a blind positioned under his chin (see Fig. 1, left panel). This was done to assess whether the lack of visual monitoring would affect his gesturing ability, as it does with instrumental actions. Not only did his gestures form cogent patterns concerning the narrative, the gesture motions seemed synchronously timed with his speech with and without visual access of his gestures, a well-known aspect of gesture-speech coordination^3,7^. The only crucially observed difference between the blind and no-blind conditions were that: (1) IW had less accuracy when his gestures required some geometric precision (or ‘topological accuracy’^9^), such as tracing an imagined triangle along its edges; and (2) gestures that mimicked object-directed actions were less likely to occur under the blind condition. It was further reported by the investigators that IW at one time seemed to have lost complete awareness that he was, in fact gesturing under the blind^10^, producing normal-looking gestures well-timed with semantically relevant portions of speech^11^. The following excerpt highlights the potential theoretical implications of IW’s gesture-speech alignment (or “morphological synchronization” as David McNeill termed it): (p. 236^7^):

“If [IW] is unable to control instrumental movements when vision is denied, but can continue to perform gestures with full accuracy and [morphological] synchronization with speech, we have demonstrated that gesture can be dissociated from action. Such a dissociation suggests the existence of a specific thought-language-hand link in the brain, distinct at some point from the pathways for controlling actions.”

Importantly, IW himself emphasizes that while he might lose track of whether he is gesturing on rare occasions, most of his gesture *initiations*, with or without vision, are carefully planned^11^. IW further reports that, in his experience, his gesturing ability is—contrary to global appearances—affected when he does not have visual access to his movements. Firstly, he reports that since he cannot sense the destabilizing forces of his gestures, he intends to produce smaller gestures without visual feedback. Secondly, he notes that control of fingers is compromised when having no visual access to his gestures. IW does acknowledge that his gesture and speech indeed operate as a single package for him, even without visual feedback. However, from our reading of IW’s phenomenological reports (Cole, 2016), he finds it difficult to describe *how* he accomplishes what indeed looks like uncompromised levels of gesture-speech synchrony.

So what do we know about gesture-speech coordination in typical subjects? Gestures do not only relate to the content of speech, also the timing of gesture relates to the prosody (and related acoustic) fluctuations of concurrent speech^3,22–27^. This synchronization of gesture with speech has shown to support the perception of speech prosody^28^. A growing body of work has shown that gesture-speech synchrony is bi-directionally coupled, such that changes in speech or gesture lead to immediate readjustments in the speech or gesture modality^22,24–26,29^. For example, when participants are asked to point and name objects in a virtual-reality environment, manipulating visual information about their hand position results in rapid adjustments to both speech and gesture. This shows that speech-gesture timing unfolds dynamically, rather than stemming from a pre-planned fixed trajectory^29,30^. It also confirms that visual information can matter for gesture control, though little is known about experimentally taking away visual access of gestures. This raises the question of how someone who lacks such continuous feedback of the body can maintain gesture-speech coupling, especially in the additional absence of visual feedback of the body. This question is made even more pertinent considering that the intersegmental timing between relevant joints in complex multi-joint actions and bimanual coordination is distorted in persons with deafferentation^31,32^, leading us to suspect that gesture-speech synchrony should be compromised too. Thus, it is unclear whether the feat of gesture-speech synchrony is somehow “distinct… …from the pathways for controlling actions” (p. 236^7^.

### Possible resources for IW’s coordination of gesture-speech synchrony

What we currently know is that IW maintains that he uses visual control for his gestures^11^. But we also know that this self-reported visual control is unavailable to him under the blind. Yet, IW further reports in one of the videos we are investigating that he is not entirely oblivious to his hand-movements under the blind. IW can at times feel temperature fluctuations from moving air particles displaced by gesture-movements, but he stresses that these are very minimal cues for the detection of movement. Movement control via air-flow sensing is also unlikely given the relatively longer time it takes for a temperature sensation to reach awareness due to low nerve-conduction velocity of temperature-related nerve fibers^33^.

What are additional information sources possibly available to IW to coordinate speech and gesture? One answer to this question is motivated by research in humans who have lost haptic perception of their upper limbs. Careful study reveals that these individuals can perform various feats of touch-based perception^34^ and sensorimotor timing^35–37^. For example, persons with haptic sensory loss in their extremities can perceive objects strapped into their hands, but only when active exploratory movements are performed. When the objects were passively held, neither the presence of an object, or basic object properties (e.g. the weight/dimensions of the object) could be determined^34^. Active exploratory movements generate forces that affect both the insensate peripheral tissues of the hands and arms, in these individuals, as well as sensorily intact tissues of the trunk and neck. By wielding the object with a limb, specific patterns of tissue deformation arise in the trunk and neck that are informative about the properties of the object being wielded^34,38^. This suggests that the cascading mechanical effects of upper limb movements onto the body can be exploited in haptic perception, and similar resources could be available to IW in orchestrating gesture and speech.

We therefore explore two related potential sources of IW’s gesture-speech abilities, similarly grounded in the idea that haptic perception and action exploits biomechanical perturbations of movements (p. 342^38^): Biomechanical gesture-respiratory-vocal interactions and biomechanical gesture-head movement interactions.

#### Gesture-respiratory-vocal interactions

Recent research on gesture-respiratory-vocal interactions revealed that physical impulses of gestures can mechanically transfer onto the respiratory system^39,40^ and can thereby directly affect key acoustic markers of speech prosody such as the Fundamental Frequency (F0; perceived as pitch) and the amplitude envelope of the concurrent vocalization^12,41–43^. Consider that the physical impulse is a force quantity determined by the change in momentum of a body segment. The change of momentum of a body segment is the change in velocity multiplied by the mass of the body segment. The change of momentum (i.e., physical impulse) can thus be increased in two ways, either by (a) accelerating (increase velocity) or decelerating (decrease velocity) rapidly, or (b) increasing the mass of the thing(s) that undergoes acceleration or deceleration. In line with the assertion that physical impulses can affect the voice, it has been found that sudden acceleration or deceleration of an upper limb segment tends to align and predict the magnitude of a positive peak in F0 or the amplitude in concurrent vocalizations^12,42^, mono-syllable utterances^41^, and even fluent speech and vocal music performances^43,44^. It has further been found that peaks in vocalization are more extreme for body segments that have a higher mass, where arm motions around the elbow generate more pronounced vocal peaks than hand motions around the wrist^41–43^. Similarly, producing bimanual movements generates more pronounced vocal peaks than unimanual motions, given that the combined mass of the body segments in motion is effectively doubled, generating more impulse per unit acceleration. These combined findings are all in line with the idea that *physical impulses* of gestures are interacting with the vocal system.

#### Gesture-head movement interactions

A related additional implication of gesture-induced forces cascading through the body is that other body parts may move in subtle ways as a consequence of those forces. Given that IW’s intact kinesthetic and vestibular perception of movement above the neck and some kinesthetic access to the lower neck region (although the C3 nerve is affected by his disease; Jonathan Cole, personal communication), we focus on gesture-correlated movement of the head which would provide detectable information about gesture. It has been observed that the presence and direction of forcefield perturbations to arm-movement trajectories can be detected without vision by persons with proprioceptive loss, including IW, and this has been shown to relate to head displacements that are mechanically affected by upper limb perturbations^45^. Since head displacements may even mechanically perturb the vocal system by changing vocal tract postures possibly affecting acoustic qualities of vocalization^46–48^, head motions may be another potential route for gestures to tune vocalization.

### Implications of a biomechanical link between gesture and speech

Finding evidence for biomechanical gesture-speech interactions would call into question the assertion that there is no information available about gesture to IW. In terms of control, if IW vocal functioning is affected by gesture, or head movement perturbations result from gesture, then there might be some more or less implicit sense available to IW about whether a produced gesture is misaligned with the speech target. This feedback can then help him optimize his gesture and speech initiations to be more synchronized. Thus, biomechanical *feedback* can optimize *feedforward* control. Interestingly the vocal apparatus is likely sensorily intact in IW given its innervation via the cranial vagal nerve (Cole, personal communication), thus possibly providing some direct access of physical impulses^49,50^. More generally, a biomechanical link means that gesture-speech synchrony does not bypass feedback control pathways that are essential in non-communicative action. As such we should not see communicative hand movements as qualitatively promoted forms of everyday action that are regulated entirely by linguistic domain-specific mechanisms. Indeed, synchronization through biomechanics is a pervasive phenomenon across a range animal taxa^51–54^; e.g., flying bats synchronize their echo-vocalization pulses with their wing beats due to perturbations on the respiratory system^53^.

### Present study

To summarize: IW lacks proprioception but maintains gesture-speech synchrony without vision while actions break down. Thus actions and gesture-speech utterances may be controlled differently in terms of visual control (general research question 1). Further, gesture-speech synchrony may unfold via other motor control resources having to do with biomechanics (general research question 2). These research questions are not mutually exclusive, as both visual control and biomechanics may help guide gesture-speech synchrony in IW.

#### General research question 1: Does visual control matter for gesture-speech synchrony for IW

The original descriptive research did not focus on the micro-scale synchronization between gesture and speech^7^, but rather addressed whether the content of gestures was matching and roughly temporally aligned with the content of the speech (i.e., morphological synchronization). In the current study, we reanalyze data from the 1998 and 2002 experiments with modern quantitative analyses of gesture kinematics and speech acoustics that were not available at the time of the original experiments. We extract the 2D body-movement trajectories from the video materials using OpenPose^55^ in conjunction with acoustic analyses^56^, see Fig. 1.

We assessed the degree of gesture-speech coordination, produced with and without a blind positioned under the chin, by computing the timing of the gesture’s peak speed relative to the peak in F0 of the nearest syllable^3,24,57^. If visual control plays no role in gesture-speech synchrony, we would not expect the gesture-speech timing distributions to be statistically different. If IW uses visual control to maintain gesture-speech synchrony, we would expect much higher variability in the gesture-speech timing distributions for the blind versus the no-blind condition.

#### General research question 2: The role of biomechanics as an additional means of motor control of gesture-speech synchrony

We hypothesized that gesture-speech coordination in IW would be associated with biomechanical coupling between the actions producing gestures and vocal utterances. Given that moments of peak impulse in a gesture have recently been shown to be associated with higher acoustic outputs^12,41,43^, and that the physical impulse of a gesture increases with limb deceleration and the mass of the effector(s), we predict that prosodic markers of speech (F0 and amplitude envelope) will depend upon: (1) gesture deceleration magnitude, and (2) whether gesturing was unimanual or bimanual. We will then also assess whether there might be a reorganization of the gesture-speech dynamics, such that synchronization through biomechanics becomes more apparent under the blind; e.g., by recruiting more forceful gesture decelerations under the blind vs. the no-blind condition. We also predict that mechanical loading of the gesture can affect displacement of the head due to mechanical loading onto the body, possibly allowing IW to detect accelerative moments in upper limb movements via sensorily intact head movements^45^. Finally, we compare different ways of quantifying synchronization (peak F0-peak speed vs. peak F0-peak impulse) to further probe whether there is a reorganization of gesture-speech coordination.

## Method

### Participant, task design and procedure

The Institutional Review Board of the University of Connecticut approved the current study (#H10-040). IW (who is left-handed) was tested at ages of 46 and 50 in two experiments, one performed in 1998 and another in 2002^9,21^. The video-footage that we obtained included IW retelling the Tweety and Sylvester cartoon ‘canary row’ (1998 experiment) and ‘snow business’ (2002 experiment), as well as talking to the experimenters about his phenomenology of gesturing under and without the blind during the 1998 experiment. One video was excluded for the current gesture-speech analyses because IW was demonstrating his movements rather than producing spontaneous co-speech gestures. A portion of another video was excluded due to a damaged portion of the video track which lead to misalignments in video and audio. Table 1 shows summary information of the data that were available for this study.

**Table 1:** Information available data.

Video number	Resolution	Experiment	Experiment Setting	Condition	Duration (seconds)	Left-Hand Gestures
1	352 × 240 (29.97fps)	1998	Interview	Blind	61	7
2	352 × 240 (29.97fps)	1998	Interview	Blind	224	24
3	352 × 240 (29.97fps)	1998	Cartoon (CR)	Blind	38	11
4	352 × 240 (29.97fps)	1998	Cartoon (CR)	No blind	139	21
5	352 × 240 (29.97fps)	1998	Interview	No blind	21	3
6	320 × 240 (29.97fps)	2002	Cartoon (SB)	Blind	139	20
7	320 × 240 (29.97fps)	2002	Cartoon (SB)	Blind	273	55
				Total	895	141
				Total blind	735	117
				Total no blind	160	24

Experiment setting refers to what IW was talking about, cartoon CR = retelling ‘canary row’, cartoon SB = retelling ‘snow business’.

### Gesture annotations

The gesture annotation was performed in ELAN^58,59^ in which we identified the gesture events from the videos. We then used the time-series from the motion tracking as a visual aid to determine the start of the gesture and the end of a gesture. Similar to other motion-tracking studies on gesture^24,56^, we annotated the start of gesture from the point at which the gesture initiated its main meaningful stroke^60^ to the point wherein it reaches a halt. Thus, our gesture annotations do not include a pre-stroke initiation that transports the hand from a resting position into a gesture space, nor a post-stroke hold which keeps the gesture motionless while the relevant speech is completing, nor a retraction phase which transports the hand back to a resting position after the main stroke.

### Gesture rates

All of IW’s gestures were produced by the dominant left-hand (n = 91) or included the left hand (in the case of two-handed/bimanual gestures; n = 50), except for 3 right-handed ones. Given the dominance of left-hand in IW’s gesturing rates, we only performed analyses for the left hand, excluding the three right-handed unimanual gestures. Table 2 shows the gesturing rates for the different experiments and conditions (blind vs. no blind). Although gesture typology is not of main interest in this study, we also coded roughly whether gestures were clearly representing something in an iconic or metaphoric fashion (representational gestures). If gestures did not convey clear iconic or metaphoric content they were coded as non-representational gestures.

**Table 2:** Gesture rates.

Experiment	Condition	Gesture rate per minute M (*SD*)	Gesture rate per syllable M (*SD*)	% representational gestures M (*SD*)
Year 1998	Blind	10.24 *(6.23)*	0.05 *(0.34)*	51.71% *(21.27)*
	No Blind	8.85 (*0.34)*	0.04 *(0.01)*	97.62% *(3.36)*
Year 2002	Blind	10.37 *(10.37)*	0.07 *(0.01)*	55.68% *(29.25)*

Standard deviations refer to variability across the different videos. Gesture rates with student populations show similar gesture rates with these cartoons, showing of about 10 representational gestures per minute^24^.

There are a couple important notes to make based on the results shown in Table 2. Firstly, the number of gestures performed (either normalized for time or normalized for syllable rate) is not dramatically different across blind versus no blind conditions. However, gestures performed with visual access (no blind condition) conveyed clear iconic or metaphoric content while gestures under the blind showed less representational content, only about half the time. What this suggests is that gestures under the blind are constrained in some way as compared to the no blind condition, which further motivates a fine-grained analysis of kinematics and acoustics.

### Motion tracking

We performed 2D videography motion-tracking using OpenPose^55^ which provided us with pose estimates resampled at 30 Hz. We extracted the left-wrist (for physical impulse analysis) and left-index-finger (for timing analysis) position data. We then applied a zero-lag second-order 10 Hz low-pass filter to smooth position (x, y) data. Smoothing parameters were chosen so as to minimize jitter in the time series (which were at times prominent given the low resolution of the video data). We used ELAN^58,59^ to continuously reassess time series and smoothing parameters together with the audio-visual-recordings. From the position data, we calculated the first- and second time derivatives (speed, acceleration) wherein we applied a zero-lag first-order 33 Hz low-pass Butterworth filter. Importantly, we z-normalized the speed and acceleration for each experiment (1998 vs. 2002). In this way, inherent differences in kinematic estimations due to camera angle differences are normalized for each experimental setting.

### Speech acoustics

We extracted audio tracks from the original video data and used PRAAT to estimate F0 at 100 Hz sampling rate. F0 is the fundamental frequency of voiced speech, an acoustic feature that is perceived as pitch. We set the F0 range to be suitable for males (75–500 Hz). The estimated mean F0 of IW was 114.99 Hz (95% CI [114.78, 115.20], *SD* = 23.23).

We also extracted a smoothed amplitude envelope (ENV) from the sound sampling at 100 Hz. The amplitude envelope captures key rhythmic components of speech as it closely relates to the syllable cycling in speech. A Praat script was used to obtain the amplitude envelope^61^, which applies a Hilbert transformation of the waveform, yielding the analytic signal from which the complex modulus is taken, which is then smoothed with a 5 Hz Hanning window. This results in a 1D smoothed amplitude envelope time series tracking gross changes in the amplitude of the waveform, and is strongly related to labial kinematics during speech^62^.

### Data processing and analyses

For further data processing, we used comparable procedures as reported in^56^. With a custom-made R script, we merged speech, annotation, and motion-tracking data into single time-series dataset with 100 Hz sampling rate. To do this, we up sampled the motion-tracking data from 30 to 100 Hz through linear interpolation. From this merged dataset, we performed the following analyses.

### Timing analysis

**Figure 2:**
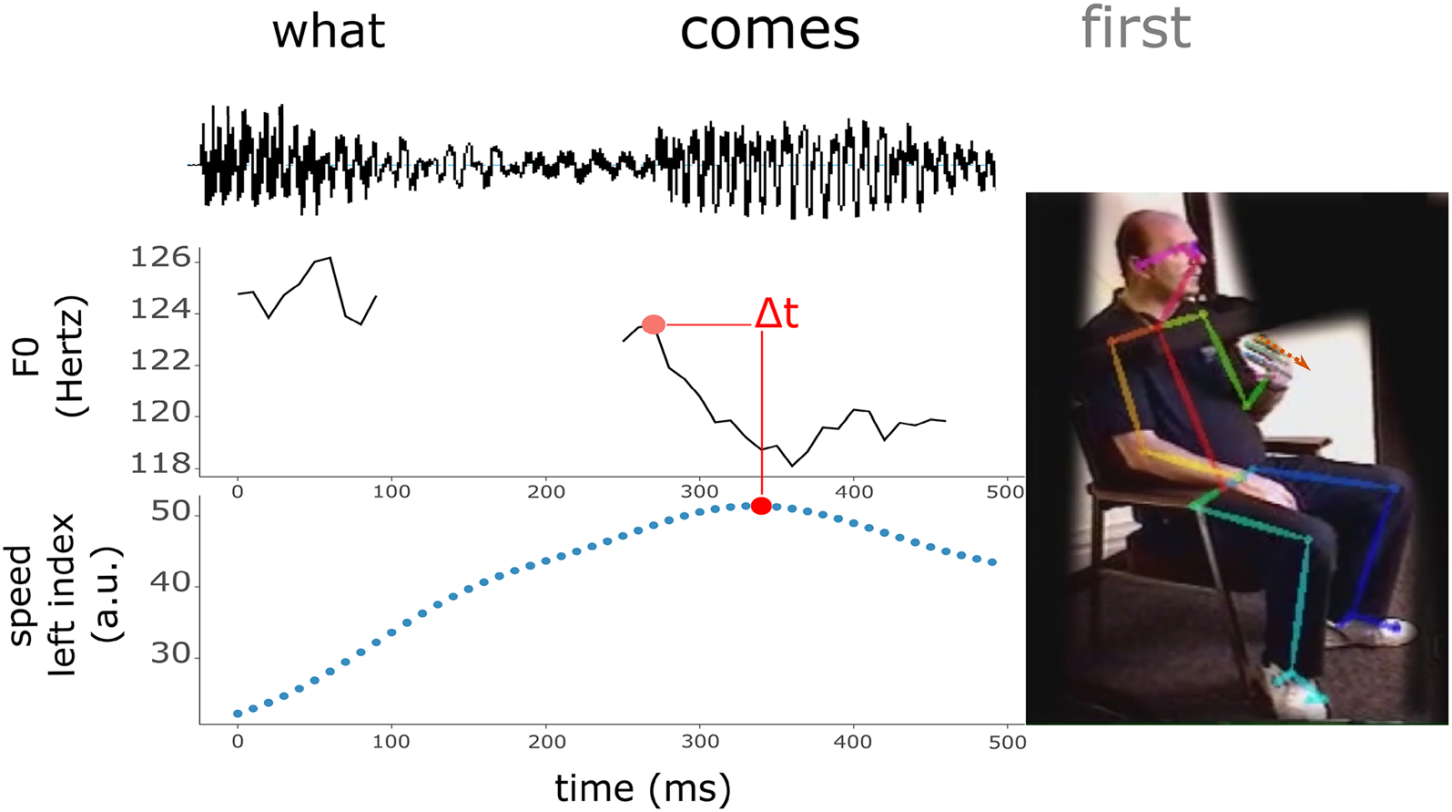
Gesture-speech synchrony timing estimation example. *Note.* Example of a gesture produced during the 1998 retelling of “canary row” cartoon wherein IW introduced the next scene “what comes first”, with an empathic stress on “comes”. Note that the first word has a higher peak F0. Given that this peak is further from peak speed the timing will be computed for the peak F0 nearest to it (i.e., the peak F0 of the second syllable).

For the timing analyses we combine gesture annotations, with speech acoustics and the motion tracking data described above. To quantify gesture-speech synchrony we computed for each gesture event the timing of the peak speed (i.e., undirected velocity) of the left index finger relative to the nearest peak in F0^24,63^; see Fig. 2. Peak speed is used because in previous research the moment with most body movement is assumed to be the maximally expressive phase of a gesture and well aligned with speech emphasis^3,57^. Using the left index finger as the gesture reference point, ensures that all joint motions (arm, wrist, index finger) are taken into account. For determining nearest peak in F0, we first determined the peak in F0 for every uninterrupted run of F0 (i.e., single phonation events). Uninterrupted runs of F0 are a proxy of syllable events, and yield the phonation part of each syllable. Subsequently, the nearest F0 to the moment of peak speed of the gesture was used to compute gesture-speech timing differences. We also performed this same timing analysis with a different kinematic point, namely the peak deceleration of the left wrist, so as to relate this to our physical impulse analysis described below. Note that we use the wrist as a gesture keypoint for analyses related to physical impulses, as motion of this joint entails that at least a segment of the arm is moving. Arm motions are more impactful than small hand movements, and thus it will allow us to more directly identify high-impulse episodes of gesture.

### Physical impulse analysis

**Figure 3:**
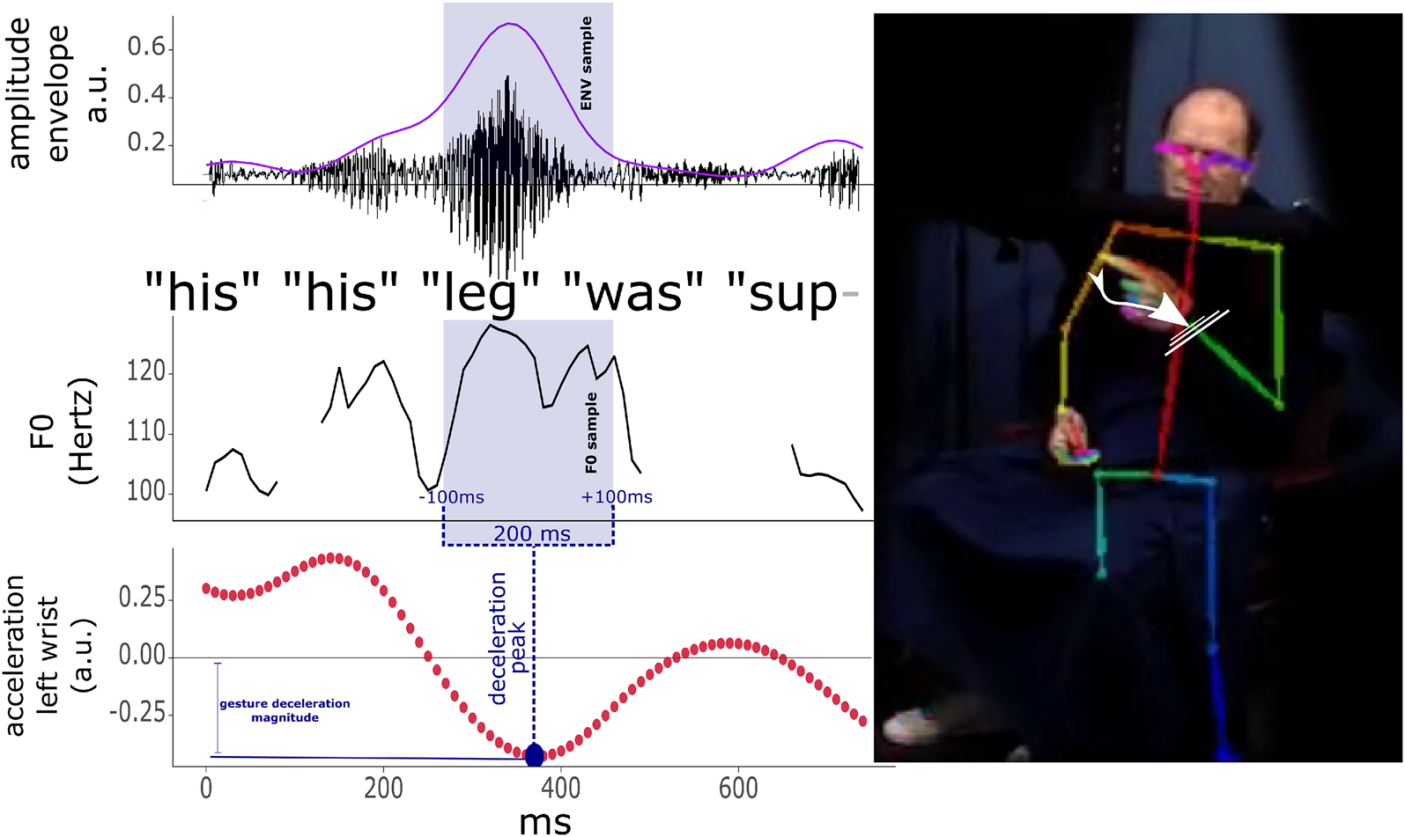
Physical impulse and acoustic analyses example. *Note*. Example of a gesture produced by IW under the blind during the 2002 retelling of the “snow business” cartoon. IW produces a gesture to signify the leg of Sylvester serving as the beam of a rotisserie. This gesture traces out the rotisseries structure but also has a quite sudden halt or peak in deceleration. This gesture’s deceleration rate was in the high impact range as compared to the other observed deceleration rates in the current data. For our physical impulse analyses, the peak deceleration was determined and then within a 200 ms interval F0, amplitude envelope (in purple), and head displacement values (not shown here) were sampled around that point. We would expect more extreme decelerations (higher impulse gestures) would coincide with observations of F0 and the amplitude envelope.

For the physical impulse analyses, we also combine gesture annotations, with speech acoustics and the motion tracking data described above. Figure 3 shows the procedures for our physical impulse analysis. For this analysis, we determined for each gesture event: (a) the moment of the peak in deceleration of the left wrist, (b) the value of the deceleration rate at that peak, and (c) the observed F0 and amplitude envelope (ENV) values within + 100 and − 100 ms around the peak in deceleration (i.e., 200 ms interval range). Earlier research has shown that at peaks of physical impulse of a gesture, we should expect increased F0 and ENV at about 70–100 ms before and after peak physical impulse^12^. Such effects were obtained for whole-arm gestures rather than finger movements and therefore we computed acceleration profiles for the left wrist rather than the index-finger which produces little physical impulse if moved on its own. We also take into account: d) whether a gesture was performed with one effector (unimanual: left hand) or two effectors (bimanual: left and right hand), though the deceleration value will always be determined based on the left wrist. According to previous research^12^, we should expect that higher physical impulse gestures (higher deceleration, bimanual gestures) have more extreme effects. We also assess: e) the concurrent 2D head displacement around peak impulse of the gesture (200 ms sample), so as to see whether higher impulses also affect displacement of the upper body parts that are perceptually available to IW.

### Outliers

We removed potential outliers for all point-estimate variables (F0 peak, ENV peak, deceleration magnitude peak, head displacement peak) defined as exceeding 3 standard deviations below or above the mean. Conclusions of the current study are not changed by retaining such outliers. However, given that OpenPose motion-tracking sometimes showed large-amplitude jitter because of the low resolution of the video material, we obtained some extreme values for deceleration in a few instances. Equivalently, PRAAT acoustic estimation of F0 with the current suboptimal audio can lead to under and overestimations.

### Ethical approval

All methods were carried out in accordance with relevant guidelines and regulations. All experimental protocols were approved by the ethical review board of the University of Connecticut. Informed consent from all subjects for publication of identifying information/images in an online open-access publication in the methods section has been obtained. We obtained informed consent from the subjects involved to participate in this experiment.

## Results

### Variability of gesture-speech synchrony (general research question 1)

If gesture-speech temporal coordination does not require visual feedback, then obstructing visual feedback of gestures should not lead to temporal differences as compared to when visual feedback is available. We will assess this assertion with respect to peak speed relative to the peak in F0. Peak speed has been identified as a potentially key kinematic anchor for gesture-speech timing peak^3,57,63^, and it reflects the moment when movement activity is heighest.

**Figure 4:**
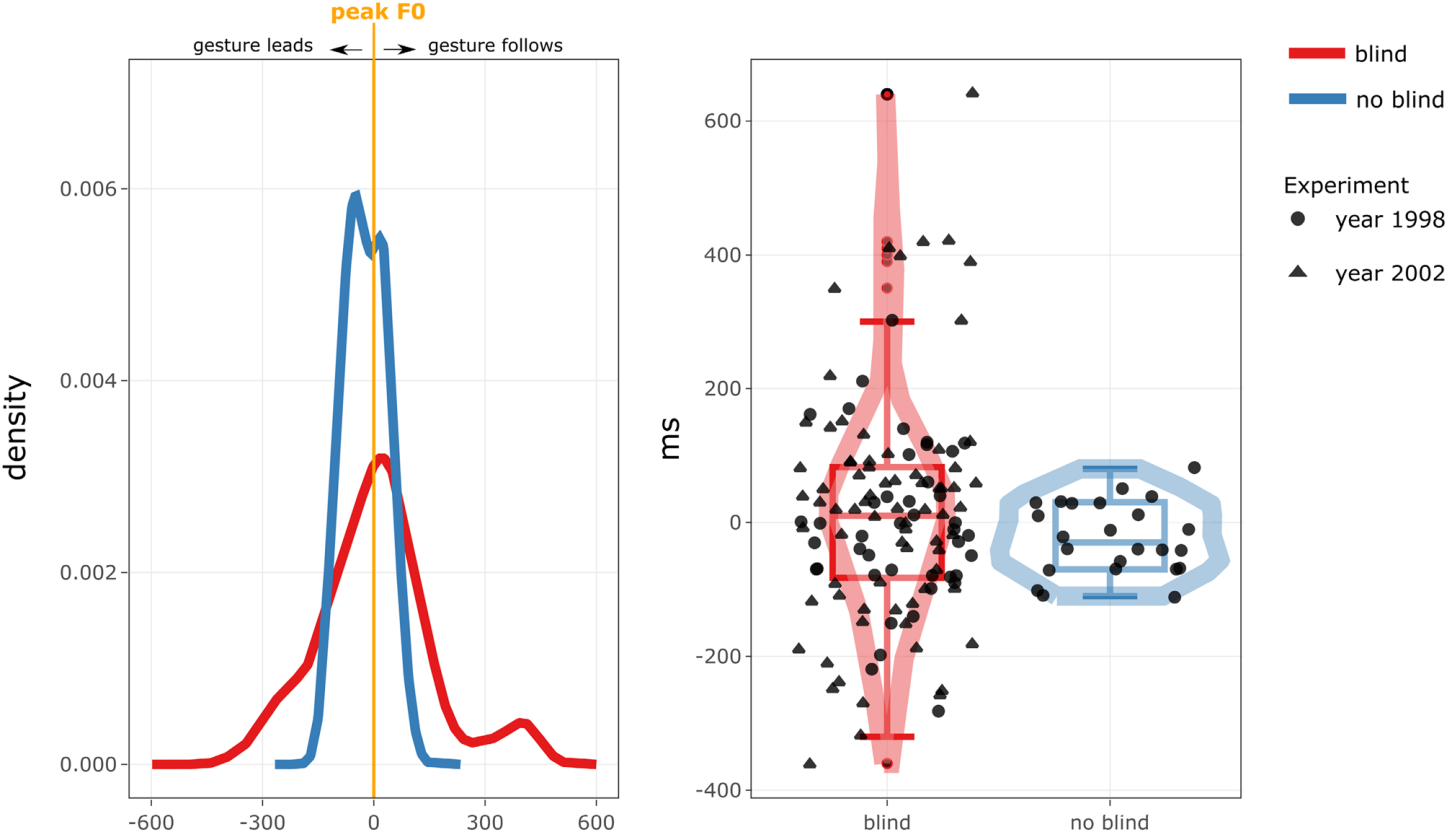
Timing between gesture peak speed and nearest peak F0. *Note*. The left panel shows the smoothed density distributions for the peak-velocity versus peak F0 timings in milliseconds (left panel: y-axis in probability units and x-axis in ms, right panel: y-axis in ms and x-axis reflecting condition). Negative (or positive) timings indicate that gesture’s peaks velocity leads (or follows) the occurrence of a nearest peak F0. The distribution widths indicate the timing variability, with larger widths showing more variable timing in gesture-speech synchrony. The right panel shows similar distribution information (violin plots) plus added information about the mean (box plots) as well as individual data points for the 1998 and 2002 experiment.

Figure 4 shows the gesture-speech timing distributions for peak speed of the left hand and the nearest peak F0, for the blind and no-blind condition. Although it should be noted that we have unequal gesture observations for the blind conditions (117 gesture events for the blind condition versus 24 for the no-blind condition), the differences in the variance of the distributions are very striking, such that variability of gesture-speech timing was much higher for the blind (*SD* = 163, mean synchrony = 11 ms) as compared to the no-blind condition (*SD* = 54, mean synchrony = − 23 ms). An *F*-test for comparing differences in variance (from the mean) of two distributions (using R function var.test; two-sided hypothesis testing) indeed revealed that observations are unlikely to have sampled from similar underlying distributions, *F*(116, 23) = 9.35 (95% CI [4.56, 16.64]), *p* < 0.001. Further note, that if we exclude the 2002 data for which we have only gestures under the blind, and compare variance distributions of only the 1998 blind vs. no blind data we obtain similar results, although with weaker effects, *F*(41, 23) = 4.715 (95% CI [2.17, 9.48]), *p* < 0.001.

In conclusion, given that we find that gesture-speech temporal coordination is much more variable when visual feedback is denied (blind condition) versus available (no blind condition), we have evidence that supports that IW uses visual information to regulate gesture-speech coordination.

### Physical impulse and acoustic markers of prosody (general research question 2)

Despite the fine-grained differences in gesture-speech synchrony for the blind and no-blind conditions documented above, it is important to reiterate that gesture-speech synchrony to the naked eye does not seem to be compromised, as originally reported^9,21^. The average gesture-speech synchrony is not very different for the blind versus no-blind condition (− 23 vs. 11 ms), and most gesture-speech asynchronies are within 300 ms of a peak F0 under the blind. The degree of asynchrony between 300 +− ms is similar to recent findings on gesture-vocal timing in neuro-typical and aphasic persons that were of a similar older age of at least 40 years old^64^. Given that IW’s gesture-speech coordination does not seem out of the ordinary, it is crucial to further probe how gestures under the blind are still able to couple reliably with speech.

We hypothesized that IW’s gesture-speech synchrony depends upon biomechanical coupling between the actions of speech and gesture, and that these biomechanical effects depend upon the physical impulse of gesture^12^.

The deceleration values obtained for each gesture were heavily positively skewed (see Figure A: https://osf.io/9k56t/). Because the relationship between end-point kinematics and the multi-joint kinetics that produce them are strongly non-linear^65^ and our measurement of kinematics are not a direct reflection of the 3D kinematics, but a 2D projection (at different angles for experiment 1998 and 2002), we used the rank-orders of the absolute-values of deceleration as an index of physical impulse.

**Figure 5:**
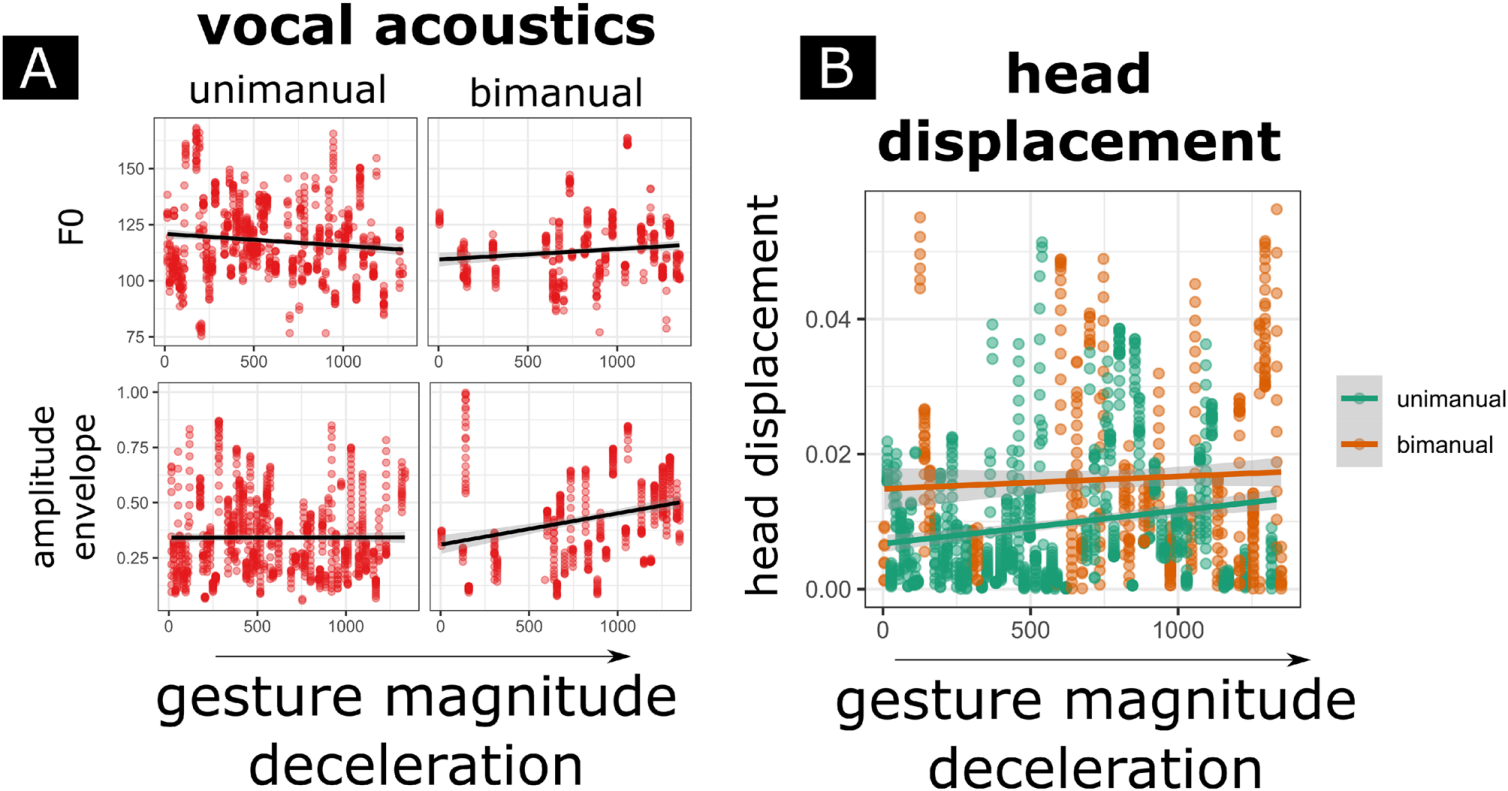
Acoustic and head displacement correlates with physical impulse. *Note*. On the x-axes, the magnitude of the deceleration (rank ordered absolutized deceleration) is given. For (**A**), the acoustic samples 200 ms around peak deceleration are plotted on the y axes, for F0 (upper; y-values in Hz) and amplitude envelope (lower; y-values in arbitrary units) subpanels. It can be seen that for bimanual gestures, a higher gesture deceleration magnitude relates to a higher acoustic output for F0 and amplitude envelope. (**B**) The relation between gesture deceleration and the displacement of the head around moments (same 200 ms sample) of peak deceleration. Head displacement is more pronounced for higher deceleration, especially for unimanual gestures. Further, bimanual gestures have in general more pronounced head displacement as indicated by the higher intercept as compared to unimanual gestures.

Figure 5 and Table 3 provide an overview of the main results. For F0 and amplitude envelope, we find that higher deceleration magnitudes for bimanual gestures relate to higher concomitant F0 and amplitude envelope output. This suggests that greater impulses (greater mass effector and higher decelerations) can be related to acoustic activity around those moments of impulse. However, this interpretation for unimanual movements is not very convincing as indicated by the left panels of Fig. 5A, even despite that our mixed regression analysis does suggest a main effect of deceleration magnitude. Note though that we further found that head displacement occurring near gesture’s peak decelerations is positively related to gesture-deceleration magnitude. Further, head movement was more extreme for bimanual rather than unimanual gestures.

**Table 3:** Acoustics and head displacement in relation to physical impulse for the blind condition.

F0	*b*	*t*(1435)	*p*
Intercept	123.965	42.14	< .001
Deceleration	− 0.0067	− 4.603	< .001
Unimanual versus bimanual	− 11.96	− 5.873	< .001
Deceleration*bimanual	0.006	2.454	< .014

Amplitude envelope	*b*	*t*(1435)	*p*
Intercept	0.3853	9.717	< .001
Deceleration	− 0.00002	− 1.218	0.223
Unimanual versus bimanual	− 0.0392	1.733	0.083
Deceleration*bimanual	87e−5	3.181	< .002

Head displacement	*b*	*t*(1263)	*p*
Intercept	0.0074	12.15	< .001
Deceleration	3e−6	4.678	< .001
Unimanual versus bimanual	0.0058	8.315	< .001

Mixed regression models with F0, Amplitude Envelope, and Head displacement as DV’s, experiment (1998, 2002) as random intercept. We modeled bimanual (vs. unimanual) gesture together with gesture deceleration magnitude, and if reliable, the interaction between bimanual gesture and deceleration magnitude (this interaction was not reliable for head displacement). Results show that bimanual gesturing moderated the effect of gesture deceleration magnitude on acoustics (F0 and Amplitude Envelope), and a higher manual gesture deceleration magnitude was related to more concomitant head displacement.

Note, that if we perform these same analyses for the no-blind condition, the manual and head physical impulses are not statistically related to acoustic output (see supplemental Table S1). This suggests that the means of synchronization might be different, inviting further analysis about whether physical impulses are more likely to be recruited in the blind condition versus non-blind condition.

### Physical impulses as resources for gesture-speech coordination (general research question 2)

**Figure 6:**
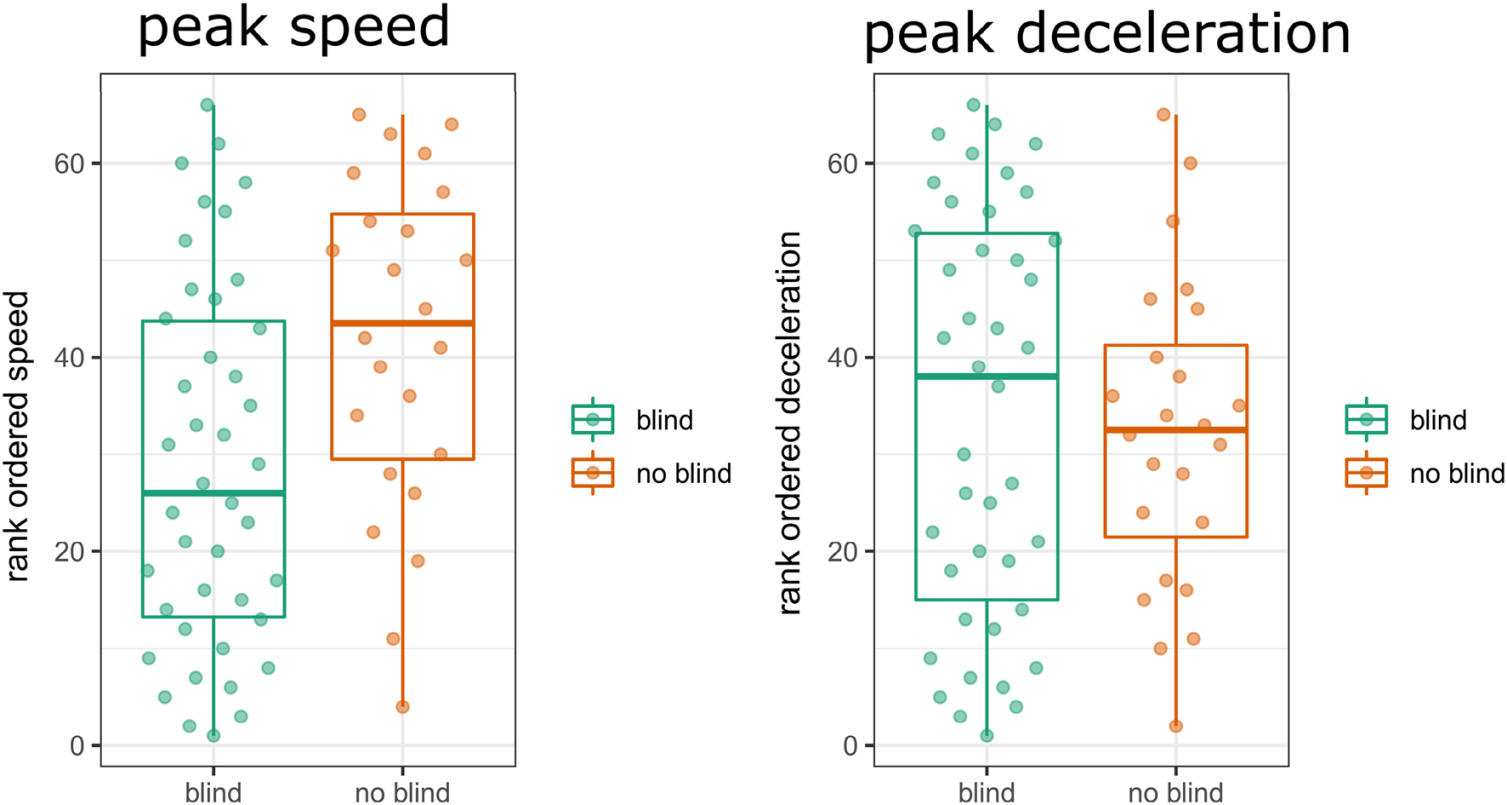
Differences in peak speeds and peak deceleration per condition. *Note*. The left panel shows the observed rank ordered peaks in speed for the blind versus the no blind condition, and the right panel shows this for peaks deceleration.

It is possible that IW is recruiting physical impulses as an anchor for synchronization when he cannot use the deliberate visual control of his gestures. If so, then we would predict higher gesture decelerations for the blind vs. the non-blind conditions. Further possibly higher speeds for the no blind condition may be observed, as under the no-blind condition the peak velocity was much more strongly coupled with peak F0. To assess this, we only look at kinematics for the 1998 experiment for which there was both blind and no-blind data. We again rank ordered the deceleration peaks as well as the speed peaks, and then compared the differences in condition. Figure 6 shows indeed that the peak speeds that were generated under the no-blind were much higher than the blind condition. But for the deceleration peaks, higher magnitudes we observed for the blind vs. no blind condition.

To assess this, we performed a mixed regression with gesture event as random intercept (as we want to compare within a gesture event two related kinematic magnitudes), and peak kinematics as dependent variable. As independent variables we have kinematic type (speed vs. deceleration) and the blind condition, as well as their interaction. The results show (Table 4) that there was indeed a reliable interaction effect, such that higher peak speeds were observed for the no blind vs. blind condition, while higher deceleration peaks were observed for the blind vs. no blind condition.

**Table 4:** Results mixed regression kinematic peaks and blind condition.

Rank value peak kinematics	*b*	*t*(64)	*p*
Intercept	28.76	9.91	< .001
Kinematic type: speed versus deceleration	5.523	2.13	.037
Blind: no blind versus blind	13.03	2.71	.008
Blind versus kinematic type	− 15.19	− 3.53	< .001

**Figure 7:**
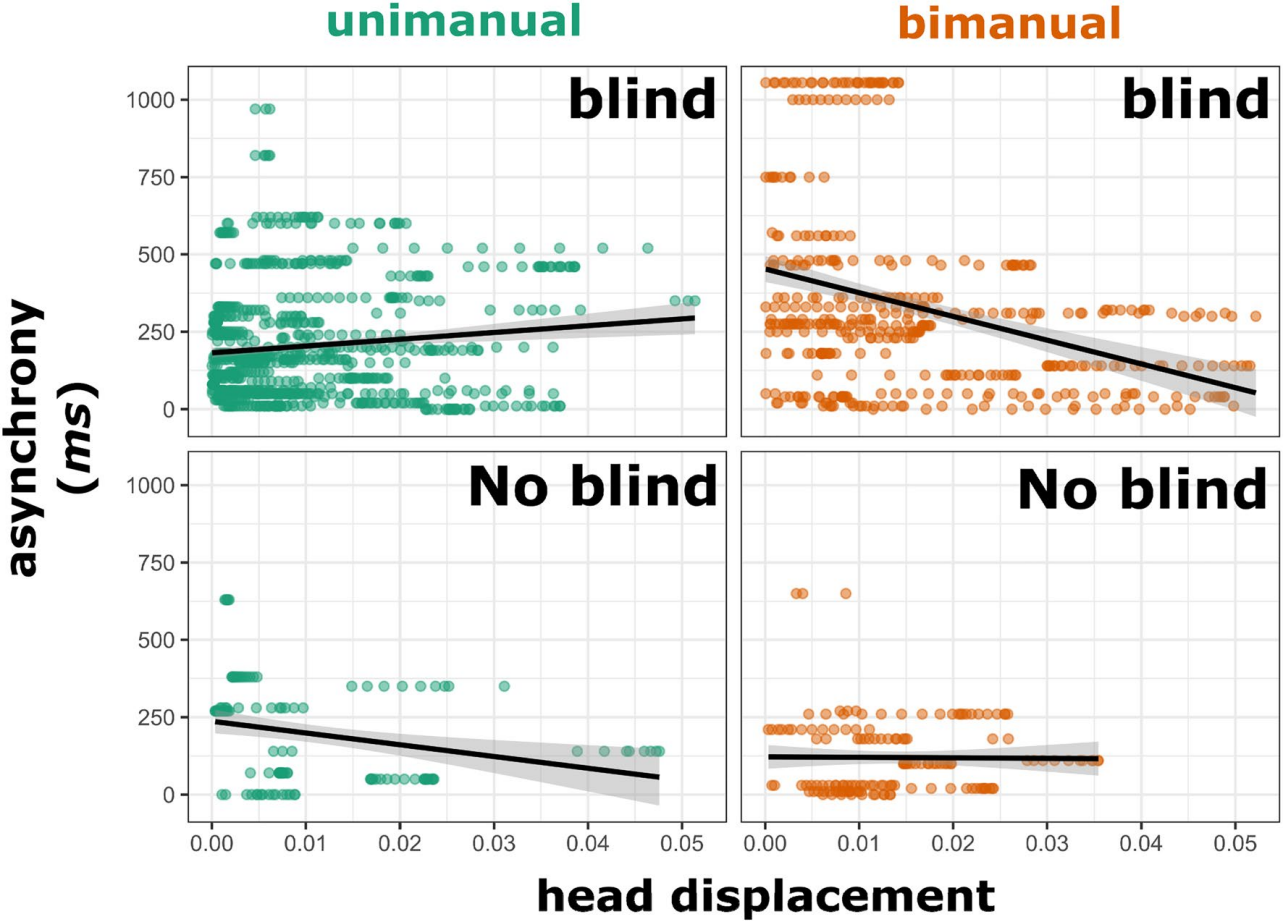
Head displacement versus asynchrony. *Note*. Head displacement sampled around a peak deceleration of the gesture is given on the x-axis (higher values mean more head displacement). Asynchrony between peak deceleration and peak F0 is given on the y axis (higher values mean more asynchrony, lower values mean more synchrony). It can be clearly seen that for the blind condition there is more synchrony when there is more head displacement, but only for higher impulse bimanual gestures. Not also that more head displacement is recruited in the blind condition as compared to the no-blind condition (in line with the idea that impulses of gestures are recruited as a resource). For the no-blind condition the relationship between head displacement and asynchrony seems to only hold for the unimanual gestures.

So far we have evidence that gestures seem to recruit more forceful movements under the blind, which affect still-sensorially-accessible head motions and are associated with acoustic markers of stress. If gesture-induced head motions are a route for closing the control loop for gesture-speech synchronization, we should predict that more head motions are associated with less asynchrony with peak F0 and peak impulse (deceleration). Figure 7 shows the main results for the blind and no blind conditions. It can be clearly seen that especially for the blind condition and for higher impulse two-handed gestures, greater head displacement was associated with less asynchrony (between F0 versus peak deceleration). We performed a mixed linear regression model (experiment as random intercept) with asynchrony as dependent variable and a three-way interaction (and their main effects) of head displacement x handedness x blind condition. We are interested in the contextual effects of head displacement on asynchrony, so we performed a post-hoc slope contrast analysis using R-package lsmeans (*p* values Tukey corrected for multiple comparisons). As reported in supplemental table S2, in the bimanual blind condition, the head displacement effect on asynchrony was reliably different from the slopes in the unimanual blind condition (*p* < 0.005), unimanual no blind (*p* < 0.001), bimanual no blind (*p* < 0.005). In the unimanual blind condition, head displacement seemed to be associated with increased asynchrony, but this slope was not reliably different from the slope in the no-blind condition bimanual condition, but only reliably different from the unimanual no blind condition (*p* < 0.005). Yet the no blind condition slopes did not reliably differ. In sum, we find some evidence that head displacement was associated with more synchrony in the bimanual blind condition, and only for this condition this effect could only be reliably contrasted with all the other conditions.

### Different sensorimotor gesture-speech solutions for the blind versus no blind condition (general research question 1 and 2)

**Figure 8:**
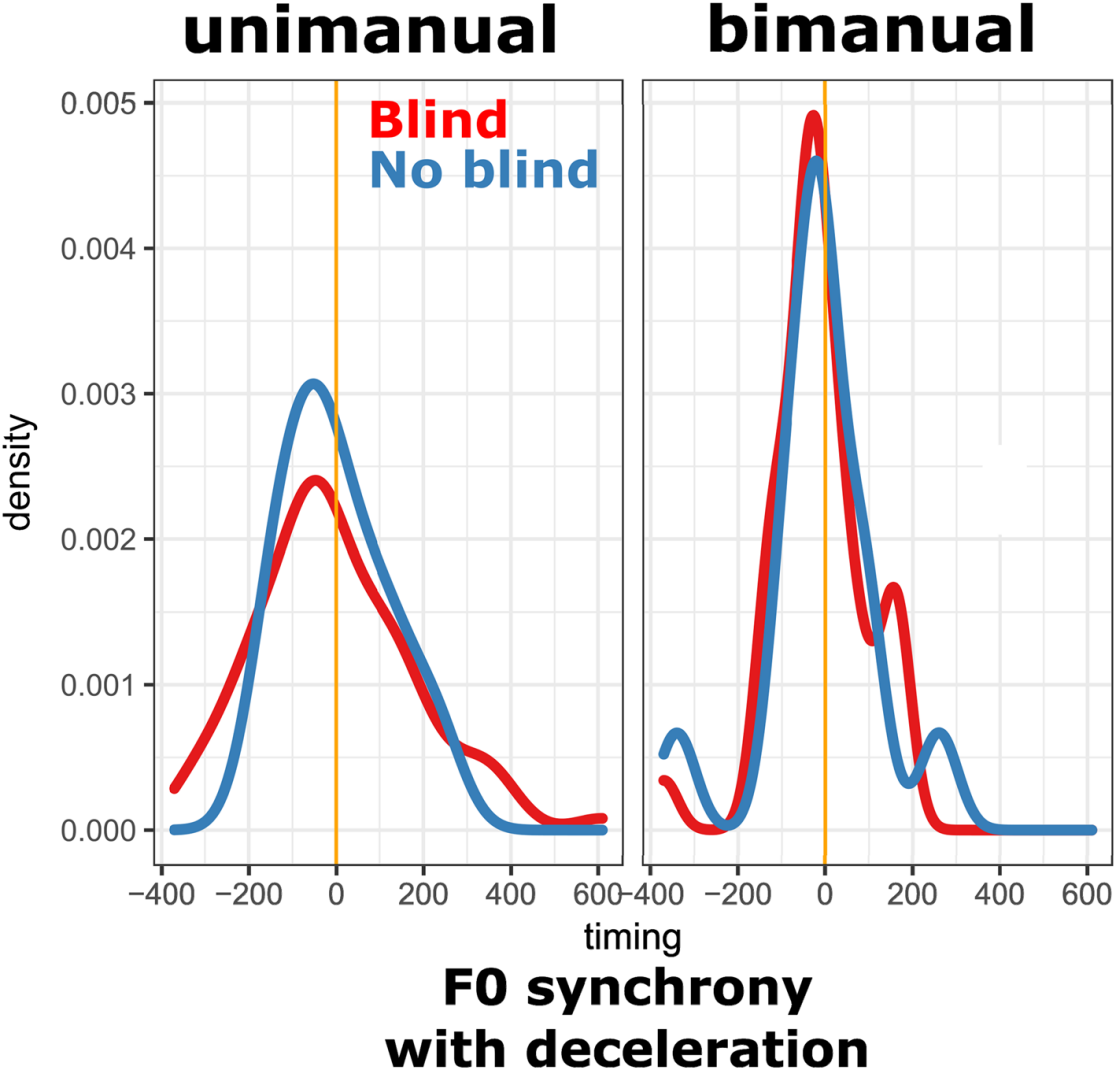
Peak deceleration and peak F0 timings. *Note*. The peak F0 versus peak deceleration timing distributions are shown, where more peaked distributions indicated more gesture-speech synchrony. It can be seen that there is a bimanual synchronization effect, suggestive of a role for physical impulses.

All the previous results seem to indicate that IW is recruiting physical impulses as an alternative sensorimotor solution for gesture-speech synchrony when visual control is not available. It is possible then the differences in timing distributions in the blind versus no-blind condition are revealing of such sensorimotor reorganization. Namely how is IW’s speech organized relative to the physical impulse (i.e., deceleration x mass of effector) or the peak in speed. Figure 8 shows the deceleration-F0 synchronization in blind and no blind condition. It shows that the differences in synchronization are not apparent between blind versus no blind conditions, both for unimanual gestures, *F*(80, 9) = 2.213 (95% CI [0.64, 5.04]), *p* = 0.196, and bimanual gestures, *F*(35, 13) = 0.657 (95% CI [0.23, 1.51]), *p* = 0.316. This seems to indicate that vocal peaks are not differently aligned with the moment of physical impulse in the blind vs. no- blind conditions. Furthermore, it was also found that bimanual gesturing in general had more synchronous physical impulse and speech coupling, *F*(90, 49) = 2.404 (95% CI [1.43, 3.88]), *p* < 0.002, supporting the idea that higher physical impulses may attract vocal modulations to align with those moments.

**Figure 9:**
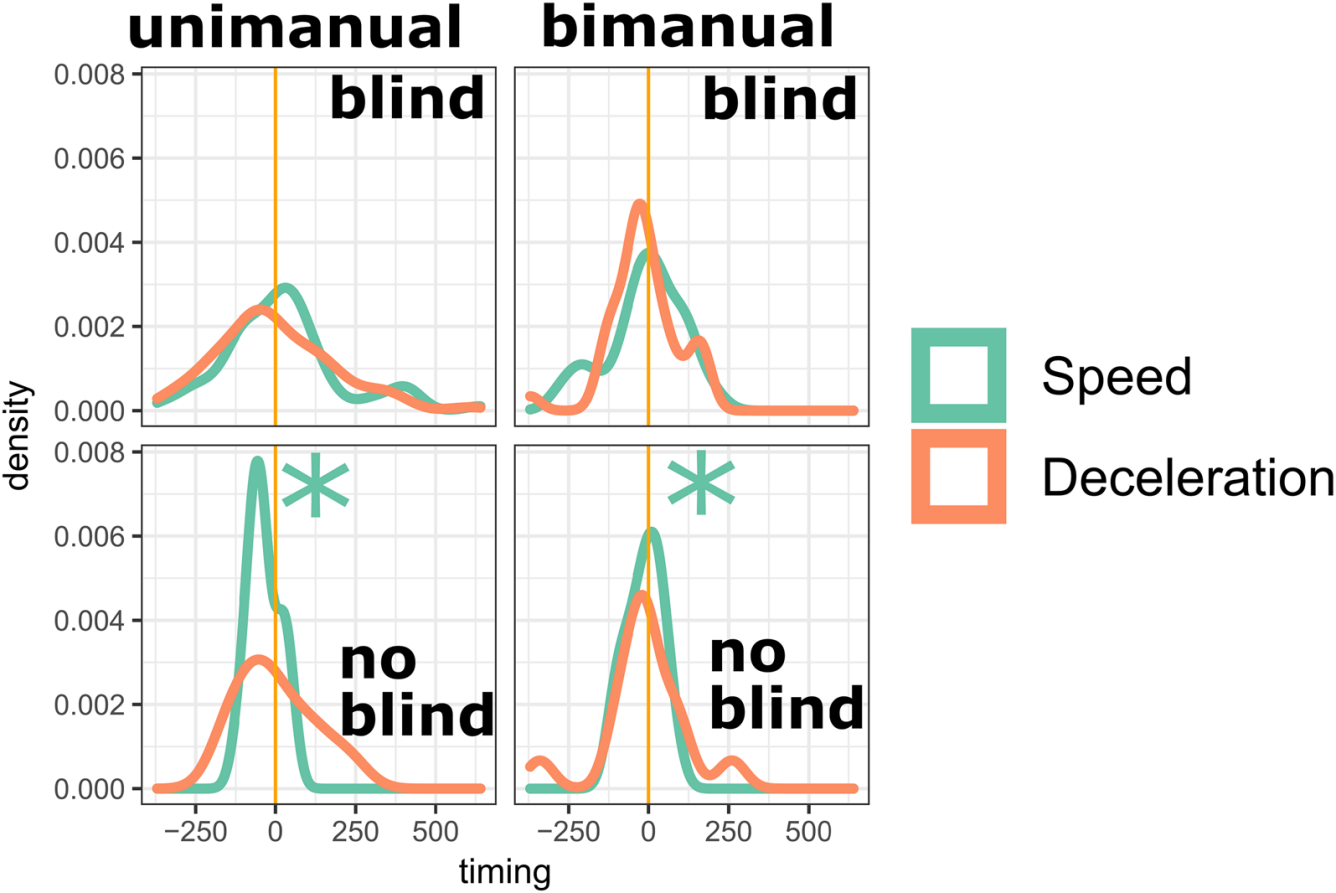
Deceleration versus speed. *Note*. Timing distributions are shown where more peaked distributions indicate better synchronization. The timings are shown for peak speed versus peak F0 (speed; in orange) and peak deceleration versus peak F0 (deceleration; green). The gestures in the no blind conditions had better synchronization for speed, but this primacy disappeared for the blind condition, providing additional evidence that there is indeed a change in the gesture-speech coordination.

There was however a salient change in the dynamics between gesture and speech depending on visual access of gesture (see Fig. 9). Namely, for the no blind condition, when visual control is available, peak speed is more synchronized with F0 then deceleration, both for the unimanual, *F*(80, 9) = 14.063 (95% CI [4.11, 32.03]), *p* < 0.001, and the bimanual gestures, *F*(13, 13) = 5.45 (95% CI [1.75, 16.96]), *p* = 0.004. However, for the blind condition, this no longer holds, and peak speed was no longer better synchronized with (*p*’s > 0.568).

## Discussion

Our analyses indicate that IW’s gesture-speech coordination is more tightly coupled to peak speed of his gestures when he has visual access to his upper limbs versus when he gestures under the blind. The differences in gesture-speech timing were impressive, showing more than 2 times greater variability under the blind. IW’s impression that visual information is key for the coordination of his gestures^11^ is consistent with our results. However, we uncovered what seems to be an additional sensorimotor solution to maintain gesture-speech synchrony, which can explain why his macro-scale gesture-speech synchrony seems largely unaltered as reported in previous seminal studies^9,10,21^.

We hypothesized that IW’s impressive gesture-speech synchrony under the blind could be explained, in part, by the biomechanical forces generated by gesture and their impact across the body. Specifically, we assessed whether the degree of physical impulse, approximated by the rate of limb deceleration and whether gestures were unimanually or bimanually performed, scales with levels of F0 and amplitude envelope (ENV) at moments when a gesture reaches such peak impulse. We found that F0 and ENV were related to the degree of physical impulse, although only bimanual gestures showed reliable increases in F0 and amplitude envelope for higher decelerated gestures. We further showed that deceleration magnitude of the gesture related to the concurrent displacement of the head around peak impulse. IW further recruited more forceful gestures under the blind condition, as indicated by higher deceleration peaks, while in the no blind condition, peaks in speed seemed to be more prominently recruited. This indicates that there is some kind of change in the gesture-speech dynamics in the blind vs. the no-blind condition, where synchronization through peak impulse is more prominent in the blind condition. We indeed found that head displacement was related to the degree of gesture-speech asynchrony between peak F0 and peak deceleration of the hand. We also showed that peak F0 relative to that peak in physical impulse is as synchronized with speech, as compared to peak in speed in the blind condition. In the no-blind condition, the coordination seems to switch to a speed-F0 coordination. Finally, we showed that higher impulse bimanual gestures were better synchronized with in terms of deceleration-F0 synchronization, suggesting a role for physical impulses in gesture-speech coordination in the blind condition.

Extending previous descriptive research performed with IW, we find that visual feedback affects micro-scale synchronization. This means that visual control can matter for gesture when proprioception is compromised; and this does not necessarily mean that visual feedback plays a major role in gesture control in typical subjects (but see^29^). In addition, we offer support for a new hypothesis about how gesture-speech synchrony can occur when IW cannot see his hands. Originally it was reported that gestures’ spatiotemporal accuracy but not their semantic accuracy was compromised under the blind. Further, the semantic content of the gestures were well aligned in time with the semantic content of speech^9,21^. Our results suggest that there is also a reorganization of gestures’ timing with speech on the prosodic level.

On the theoretical side, we think that David McNeill’s (and colleagues) original views on IW’s astonishing gesture-speech coordination under the blind resonates with our current biomechanical hypothesis. McNeill’s growth-point theory may be summarized as the thesis that gesture and speech comprise a single multimodal utterance; The very formulation of speech is entangled with the formulation of gesture. We think this theory comes very close to our biomechanical take on the issue, given that gestures are physically co-entangled with speech through the forces reverberating across a pre-stressed system^34,38^. There are many sensorimotor loops that usually help to entangle multiple bodily processes, and it seems that IW prefers a deliberately visually controlled gesture-speech synchrony when possible, but falls back on pre-reflective resources that are always available by virtue of the body’s tensegrity structure^38,66^; a structure that allows local perturbations to reverberate globally due to being a pre-stressed system of tensile (e.g., muscles) and compressive (e.g., bones) elements (for a detailed discussion see Pouw & Fuchs^67^)﻿. Interestingly, it has been said that IW has lost his body in some sense^8^. In another sense, his regained ability to gesture, even without visual access, constitutes a rediscovery of the body’s potential for meaningful expression. This view resonates with, for example, Susan Hurley’s classic analysis^68^ of split-brain patients who despite a neural detriment do often act as a single intentional system. In her view, this is because there are “dynamic causal loops” that involve processes distributed over body and environment which reconnect otherwise unconnected neural hemispheres. The current findings suggest that biomechanics offer IW dynamic causal loops that allow for coordinating gesture and speech trajectories without visual access.

The differences we observed in the way IW synchronized gesture and speech suggests that indeed when vision is available there seems to be an effort to time visual motion intensity (speed) with prosodic markers (peak F0). However, when this is not available, another source of timing is exploited. The physical intensity (physical impulse) is “timed” with prosodic markers through the mechanical loading of high-impulse gestures onto the upper trunk musculo-skeletal system which are known to increase lung pressure^41–43^. These physical impulses thereby constrain the vocal apparatus. We have further shown that head displacements were related to high impulse gestures and increased gesture-speech synchrony, suggesting that the upper body is perturbed by gesturing and these perturbations provide a resource for IW given intact vestibular sensations and proprioception above the neck. Though, we should emphasize that our mechanical explanation is not experienced by IW (Cole, personal communication). Our analyses further revealed that physical impulses of a gesture were related to the speech acoustics. Higher impulse gestures might thus be a biophysical means to align movement with speech.

How IW produces manual communicative movements has been a mystery, and aspects of his performance have been explicable only through language-specialized neural-cognitive mechanisms that bypass **“**pathways for controlling [instrumental] actions”^2^ [p. 236]**.** In the current research, we have obtained results that may demystify IW’s gesture-speech timing, showing that vision and biomechanics are resources for orchestrating gesture and intonation. These findings suggest that gestures are not necessarily based on a special language-dedicated mechanism that implies completely different means of motor control relative to object-directed actions. The bodily resources for controlling actions may just be more diverse than generally appreciated. That IW is able to use such resources in complex communication remains a marvelous achievement of action and perception.

## Supplementary Information

Supplementary Tables.

## Data Availability

Raw quantitative data, and analysis scripts supporting this study are available on the OSF (OSF: https://osf.io/q98n5/).
