# Supplementary material for: The importance of visual control and biomechanics in the regulation of gesture-speech synchrony for an individual deprived of proprioceptive feedback of body position
Source: Sci Rep. 2022 Aug 30;12:14775. doi: 10.1038/s41598-022-18300-x (PMC9428168; doi:10.1038/s41598-022-18300-x)
Supplement: Supplementary file 1 — Supplementary Tables. [file 41598_2022_18300_MOESM1_ESM.docx]

**Supplemental Materials**

Table S1. Acoustics and head displacement as predicted by physical impulse for the no blind condition

| **F0** | *b* | *t*(274) | *p* |
| --- | --- | --- | --- |
| Intercept  Deceleration  Unimanual vs. Bimanual  Deceleration*Bimanual | 129.28  -0.115  -3.185  0.004 | 17.397  -1.451  -0.393  0.578 | <.001  .148  .694  .564 |
| **Amplitude Envelope** | *b* | *t*(274) | *p* |
| Intercept  Deceleration  Unimanual vs. Bimanual  Deceleration*Bimanual | 0.429  -0.00001  0.058  -0.0001 | 7.344  0.298  0.911  -1.513 | <.001  0.766  0.363  .132 |
| **Head displacement** | *b* | *t*(259) | *p* |
| Intercept  Deceleration  Unimanual vs. Bimanual | -0.144  -.5e5  .0042 | 9.073  -3.823  3.975 | <.001  <.001  <.001 |

*Note*. These are the same analysis as reported in table 3, but now only for gestures produced for the no-blind condition. Note that, the effects are not statistically reliable, or are in the opposite direction, as compared to the primary results on the blind condition.

Table S2. Trends and contrast between trends for head displacement and asynchrony

| **Condition** | ***b head displacement***  ***95%CI[lower, upper]*** | ***SE*** |  |
| --- | --- | --- | --- |
| Blind unimanual  Blind bimanual  No blind unimanual  No blind bimanual | 2229  [818, 3639]  -7726  [-9189, 6264]  -3793  [-6905, 682]  -160  [-4186, 3866] | 818  746  1586  2052 |  |
| **Contrasts *lsmeans***  Blind unimanual – no blind unimanual  Blind unimanual – blind bimanual  Blind unimanual – no blind bimanual  No blind unimanual – blind bimanual  No blind unimanual – no blind bimanual  Blind bimanual – no blind bimanual | ***contrast estimate***  *6022*  *9955*  *2389*  *3933*  *-3634*  *-7566* | ***t* ratio (1525)**  3.46  9.60  1.09  2.24  -1.40  -3.47 | ***p***  *.003*  *<.001*  *.691*  *.112*  *.499*  *.003* |

*Note.* P-values for head displacement~asynchrony slope contrasts are Tukey corrected.
